# Association of Neutrophil‐to‐Lymphocyte and Platelet‐to‐Lymphocyte Ratios With Three‐Vessel Coronary Artery Disease: A Case‐Control Study

**DOI:** 10.1002/hsr2.72173

**Published:** 2026-04-28

**Authors:** Ladan Ayazkhoo, Mohammad Sistanizad, Mohammad Haji Aghajani, Roxana Sadeghi, Zahra Jafari, Niloufar Taherpour

**Affiliations:** ^1^ Department of Clinical Pharmacy, School of Pharmacy Shahid Beheshti University of Medical Sciences Tehran Iran; ^2^ Prevention of Cardiovascular Disease Research Center Shahid Beheshti University of Medical Sciences Tehran Iran; ^3^ School of Pharmacy Shahid Beheshti University of Medical Sciences Tehran Iran; ^4^ Prevention of Cardiovascular Disease Research Center, Department of Epidemiology, School of Public Health and Safety Shahid Beheshti University of Medical Sciences Tehran Iran

**Keywords:** biomarkers, coronary artery disease, inflammation, neutrophil‐to‐lymphocyte ratio, platelet‐to‐lymphocyte ratio, risk stratification

## Abstract

**Background and Aims:**

Coronary artery disease (CAD) is a leading cause of mortality worldwide, with inflammation playing a crucial role in its pathogenesis. The neutrophil‐to‐lymphocyte ratio (NLR) and platelet‐to‐lymphocyte ratio (PLR) are emerging as potential inflammatory biomarkers for CAD severity. This study investigates the association of NLR and PLR with three‐vessel disease in patients undergoing elective coronary angiography.

**Methods:**

A registry‐based case‐control study was conducted on 300 patients at Imam Hossein Hospital, Tehran, Iran. Patients were randomly divided into two groups of 150: (1) CAD patients with three‐vessel disease (≥ 50% stenosis) and (2) individuals with normal coronary angiograms. Demographic, clinical, and preoperative laboratory variables were compared between groups. Univariate and multivariable logistic regression analyses were performed to determine the predictive value of NLR and PLR. The models' performance was assessed using discrimination and calibration indices.

**Results:**

Patients with three‐vessel CAD had a significantly higher prevalence of NLR ( ≥ 2.5) and PLR ( ≥ 130) than those with normal coronary arteries (*p* < 0.05). NLR ≥ 2.5 was independently associated with a 91% increase in the odds of three‐vessel CAD (OR = 1.91, 95% CI = 1.04–3.51, *p* = 0.037). Although elevated PLR was observed in three‐vessel CAD cases, its association was not statistically significant after adjusting for confounders (OR = 1.79, 95% CI = 0.93–3.41, *p* = 0.077). The models demonstrated good overall performance, with an area under the curve of 85%.

**Conclusion:**

NLR is a significant predictor of severe CAD and may serve as a cost‐effective inflammatory biomarker for risk stratification. PLR, while associated with CAD severity, may warrant further investigation. Future studies should explore additional inflammatory markers to enhance CAD risk assessment.

## 1 Introduction

Coronary Artery Disease (CAD), a leading cause of global mortality, is responsible for approximately 17.8 million deaths annually [1, 2, 3, 4]. Its primary pathology is coronary atherosclerosis, characterized by plaque accumulation and arterial narrowing [2]. Inflammation plays a pivotal role in CAD progression, with key mediators including white blood cells (WBC), C‐reactive protein (CRP), interleukin‐6 (IL‐6), and platelets [5]. Neutrophils release cytokines that recruit monocytes, promoting plaque formation, while T lymphocytes sustain local inflammation [6, 7, 8]. B lymphocytes may exhibit dual roles, and platelets contribute actively to both thrombosis and inflammatory pathways [9, 10]. Recent attention has focused on the Neutrophil‐to‐Lymphocyte Ratio (NLR) and Platelet‐to‐Lymphocyte Ratio (PLR) as simple, cost‐effective inflammatory biomarkers for assessing CAD severity and predicting adverse outcomes [1, 11]. Elevated NLR, reflecting a shift toward pro‐inflammatory neutrophil activity over regulatory lymphocyte function, is associated with increased atherosclerosis and cardiovascular risk [4, 12, 13, 14]. Similarly, a higher PLR has been linked to greater CAD burden [11]. While coronary angiography (CAG) remains the gold standard for definitive CAD diagnosis, it is an invasive and costly procedure [15, 16]. This underscores the clinical need for reliable, non‐invasive biomarkers to aid in early risk stratification and guide decisions regarding invasive testing. However, evidence for NLR and PLR remains inconsistent. Many prior studies are limited by methodological issues, including small sample sizes, lack of appropriate control groups, and failure to adjust for major cardiovascular confounders such as obesity, diabetes, dyslipidemia, and medication use [1, 12, 17]. Furthermore, factors like age and chronic comorbidities can influence these ratios, potentially affecting their diagnostic specificity [1].

To address these gaps, this study aims to evaluate the clinical utility of NLR and PLR as predictive biomarkers in patients scheduled for elective coronary angiography. Specifically, we assess their association with three‐vessel coronary artery disease (3VD, defined as ≥ 50% stenosis), while rigorously adjusting for a comprehensive set of potential confounders.

## 2 Methods

### 2.1 Study Design and Participants

This registry‐based case–control study was conducted at Imam Hossein Educational Hospital (Tehran, Iran) between July 27, 2021, and July 12, 2024. Data were extracted from the Coronary Angiography and Angioplasty Registry (CAAR), an ongoing system that collects baseline and clinical information from adult patients (age ≥ 18 years) undergoing coronary angiography or angioplasty at the hospital [18]. From the initial cohort of 3227 patients who underwent coronary angiography/angioplasty (CAG), we categorized participants based on angiographic findings. The case group comprised patients diagnosed with three‐vessel disease (3VD, defined as ≥ 50% stenosis in three major epicardial vessels) [19]. The control group consisted of patients with normal coronary arteries (no visible disease or luminal irregularities). Patients with incomplete laboratory data for NLR or PLR estimation, those with HIV, HCV, or HBV infection, and individuals using corticosteroid medications were excluded from the study.

This study was approved by the review board of the ethics committee of the vice‐chancellor for Research, Shahid‐Beheshti University of Medical Sciences, Tehran, Iran (IR. SBMU. RETECH. REC.1401.874). All participants provided informed consent before enrolling in CAAR. In addition, ChatGPT (GPT‐5.2) was used solely for language editing and did not contribute to the scientific content of the manuscript.

### 2.2 Variables and Data Extraction

Patient information was extracted from the CAAR database and comprised demographic characteristics, clinical and medication history, anthropometric measurements, angiographic findings, and preoperative laboratory parameters. Laboratory test results were obtained within 24–48 h before angiography. For patients with multiple angiograms and corresponding tests within a year, only the test result closest to the angiography date was included in the analysis.

The Neutrophil‐to‐Lymphocyte Ratio (NLR) was calculated as the absolute neutrophil count divided by the absolute lymphocyte count. Similarly, the Platelet‐to‐Lymphocyte Ratio (PLR) was computed as the platelet count divided by the absolute lymphocyte count. Both ratios were derived from differential complete blood count (CBC) results. As differential CBC data (neutrophil, lymphocyte, and platelet counts) were not automatically recorded in the primary patient registration system, the researcher manually extracted these values from the hospital's laboratory information system. This extraction was performed while blinded to the patients' angiographic outcomes. The extracted laboratory data were subsequently merged with the main CAAR database for statistical analysis.

### 2.3 Sample Size Estimation and Sampling Method

The study aimed to model factors influencing severe CAD, using the Event Per Variable (EPV) rule instead of conventional formula‐based calculations [20]. In logistic regression modeling, at least 10 cases per independent variable are required for reliable statistical inference. Given the inclusion of 15 independent variables, a minimum of 150 patients per group (total: 300 patients) was required.

Patients included in this study were randomly selected from the CAAR database in four steps:

(1) The study population was defined based on the interpretation of angiography results (completely normal, mild coronary artery disease, single‐vessel disease, two‐vessel disease, and three‐vessel disease) (total *N* = 3227).
(2) The total number of cases in the completely normal and three‐vessel disease groups was estimated (*N* = 779 with three‐vessel disease and *N* = 500 with normal angiography results).
(3) A unique code was assigned to each patient within each group.
(4) Random numbers were generated using the “rand” function in Excel, and samples were randomly selected based on the generated random numbers within each group (*N* = 200 per group).

### 2.4 Statistical Analysis

The normal distribution of quantitative variables was assessed using a histogram. For reporting quantitative variables, measures such as mean and standard deviation (SD) or median and interquartile range (Q1–Q3) were used, while for describing categorical variables, frequency and percentage (%) were employed. To compare the means of quantitative variables between two groups, parametric tests such as Student's *t*‐test were used, or in case of non‐normal distribution, non‐parametric tests like the Mann–Whitney *U* test were used. The assumption of equality of variances in quantitative variables with normal distribution was assessed using Levene's test.

To examine the difference in the distribution of grouped variables, appropriate statistical tests like the Chi‐square test or Fisher's exact test were used. In addition, to investigate the correlation between NLR and PLR values with the percentage of Left Ventricular Ejection Fraction, Spearman's correlation test was applied. The strength of correlation was interpreted using the correlation coefficient (rho) values, with a spectrum of ≥ 0.70 indicating strong correlation, 0.70–0.40 indicating moderate correlation, and less than 0.40 indicating weak (negligible) correlation [21]. Logistic regression models with univariate and multivariable levels have been utilized in this study to investigate factors associated with the outcome and control potential confounding variables. Additionally, for selecting the best variables to enter the regression models, a stepwise selection model backward approach with a significance level of less than 0.2 (*p*‐value ≤ 0.2) has been employed.

The optimal cut‐point for NLR and PLR to predict severe coronary artery disease (3VD) was determined using the maximum Youden's index (sensitivity + specificity – 1) [22]. After that, the role of NLR and PLR was examined in two separate models at the univariate (Model 1) and multivariable levels with adjustment (Model 2 and Model 3).

Finally, the best multivariable logistic regression model was fitted based on the value of Area Under Curve (AUC, ROC curve) and Hosmer–Lemeshow test. Also, validity indices of final models, such as sensitivity, specificity, Negative Predictive Value (NPV), Positive Predictive Value (PPV), and accuracy were reported. The results of the models are reported based on the Odds Ratio (OR). All statistical analyses have been conducted using two‐tailed tests with a 95% confidence level and significance level less than 0.05. The software used for data analysis was STATA 17 (StataCorp LLC, College Station, TX 77845, USA).

## 3 Results

**Figure 1:**
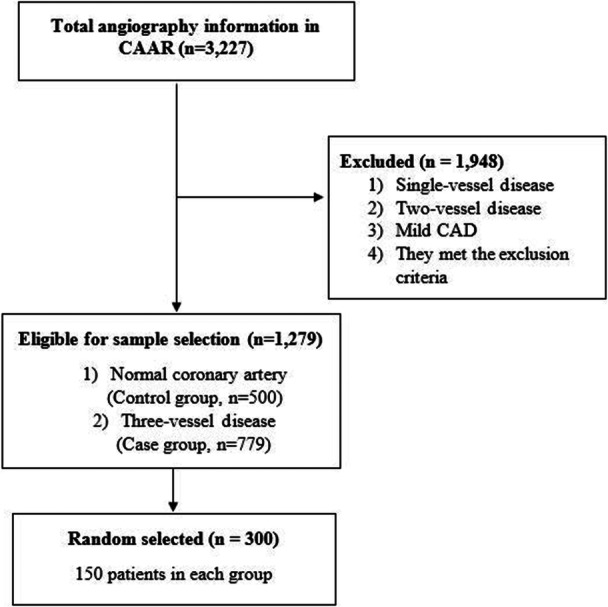
Flowchart of patients' selection.

Between July 27, 2021, and July 12, 2024, 3,227 coronary angiography or angioplasty procedures were performed. From these cases, 300 patients undergoing coronary angiography were randomly selected for further examination. These patients were then classified into a control group (n = 150, normal angiography) and a case group (*n* = 150, three‐vessel disease (3VD) with > 50% stenosis) (Figure 1).

### 3.1 General and Clinical Characteristics

The overall mean age of the patients was 57.73 ± 13.30 years, with 59.33% of the patients being over 55 years old. 163 patients (54.33%) were male. A statistically significant difference was observed in the distribution of age and gender between individuals with 3VD and those with normal results (*p* < 0.05). The prevalence of smoking in all patients was 26% (*N* = 78), and approximately 10% of the total patients reported a history of alcohol consumption.

The most common underlying diseases among individuals undergoing coronary angiography were hypertension (48.33%), dyslipidemia (39.67%), and diabetes (34%). 55.18% also reported a family history of ischemic heart disease in at least one first‐degree relative. Overall, the prevalence of the mentioned underlying conditions and a positive family history of ischemic heart disease were significantly higher in individuals with 3VD compared to individuals with normal angiography results (*p* < 0.05). Details of the patients' information are reported in Table 1.

**Table 1:** General and medical history of participants.

Variables	Normal (*n* = 150)	3VD (*n* = 150)	Total (*n* = 300)	*P*‐value
**Age (years)**	51.73 ± 12.93	63.73 ± 10.73	57.73 ± 13.30	< 0.001*
≤ 55 years	94 (62.67)	28 (18.67)	122 (40.67)	< 0.001*
> 55 years	56 (37.33)	122 (81.33)	178 (59.33)	
**Gender**				
Female	81 (54.0)	56 (37.33)	137 (45.67)	0.004*
Male	69 (46.0)	94 (62.67)	163 (54.33)	
**Anthropometric assessment**				
Waist‐to‐hip ratio (cm)	0.96 ± 0.06	0.98 ± 0.05	0.97 ± 0.06	0.004*
Body mass index (kg/m^2^)	27.72 ± 5.20	27.47 ± 4.93	27.60 ± 5.06	0.587
**Habits**				
Current smoker (Yes)	41 (27.33)	37 (24.67)	78 (26.0)	0.599
Ex‐smoker (Yes)	10 (6.67)	12 (8.0)	22 (7.33)	0.658
**Alcohol**				
No	131 (87.33)	140 (93.33)	271 (90.33)	0.035*
Yes, at least once a week ≥ 6 months	13 (8.67)	10 (6.67)	23 (7.67)	
Yes, at least once a week < 6 months	6 (4.00)	0 (0.0)	6 (2.00)	
**Medical history**				
Myocardial infarction (MI, Yes)	0 (0.0)	34 (22.67)	34 (11.33)	< 0.001*
Ischemic Heart Diseases (IHD, Yes)	16 (10.67)	65 (43.33)	81 (27.0)	< 0.001*
Cerebral Vascular Accident (CVA, Yes)	5 (3.33)	17 (11.33)	22 (7.33)	0.008*
Hypertension (Yes)	53 (35.33)	92 (61.33)	145 (48.33)	< 0.001*
Diabetes (Yes)	26 (17.33)	76 (50.67)	102 (34.0)	< 0.001*
Dyslipidemia (Yes)	34 (22.67)	85 (56.67)	119 (39.67)	< 0.001*
Chronic respiratory diseases (Yes)	10 (6.67)	10 (6.67)	20 (6.67)	1.000
Chronic Kidney Diseases (CKD, Yes)	9 (6.00)	25 (16.67)	34 (11.33)	0.004*
**Thyroid function**				
Normal	123 (82.00)	136 (90.67)	259 (86.33)	0.076
Hyperthyroidism	3 (2.00)	1 (0.67)	4 (1.33)	
Hypothyroidism	24 (16.00)	13 (8.67)	37 (12.33)	
Cancer (Yes)	3 (2.00)	10 (6.67)	13 (4.33)	0.085
Positive family history of IHD/MI (Yes)	73 (48.99)	92 (61.33)	165 (55.18)	0.032*
Acute coronary syndrome on admission (Yes)	90 (60.0)	103 (68.67)	193 (64.33)	0.117
**Drug history**				
Anti‐diabetic agents/insulin (Yes)	23 (15.33)	58 (38.67)	81 (27.0)	< 0.001*
Lipid‐lowering agents (Yes)	41 (27.33)	81 (54.00)	122 (40.67)	< 0.001*
Anti‐hypertensive agents (Yes)	65 (43.33)	97 (64.67)	162 (54.0)	< 0.001*

Data presented as mean ± standard deviation or frequency and percentage

* statistically significant, *P*‐value < 0.05

### 3.2 NLR and PLR Distribution

According to the results in Table 2, the median NLR for all participants was 2.5, with an interquartile range (IQR) of 1.68–4.16. After grouping the NLR values based on Youden's index, 60% of patients with 3VD and 40% of individuals with normal coronary arteries were in the elevated NLR group (NLR ≥ 2.5). This difference in distribution was statistically significant (*p* = 0.001).

**Table 2:** Clinical characteristics of participants.

Variables	Normal (*n* = 150)	3VD (*n* = 150)	Total (*n* = 300)	*P*‐value
Left Ventricular Ejection Fraction (%)	53 (45–55)	43 (33–50)	50 (38–55)	< 0.001*
**Laboratory results**				
Hemoglobin (g/dL)	13.7 (12.3–14.9)	13.4 (11.7–14.9)	13.5 (12–14.9)	0.367
RBC (10^6^/µL)	4.9 (4.37–5.31)	4.76 (4.21–5.15)	4.83 (4.31–5.26)	0.050
WBC (10^3^/µL)	7.9 (6.3–9.5)	8.3 (6.5–10.7)	8.1 (6.4–9.95)	0.052
Neutrophil (%)	64.1 (56–72.2)	68.75 (58.8–76)	66.65 (57.85–74.05)	0.010*
Lymphocyte (%)	28.6 (20.3–35.4)	23.6 (15.6–32.8)	26.65 (17.8–33.9)	0.001*
Platelets (10^3^/µL)	227.5 (188–267)	211.5 (174–269)	219.5 (181–267.5)	0.153
Neutrophil‐to‐Lymphocyte Ratio (NLR)	2.22 (1.57–3.56)	2.85 (1.75–4.63)	2.5 (1.68–4.16)	0.002*
< 2.5	90 (60.0)	60 (40.0)	150 (50.0)	0.001*
≥ 2.5	60 (40.0)	90 (60.0)	150 (50.0)	
Platelets‐to‐Lymphocyte Ratio (PLR)	108.40 (84.09–136.46)	113.37 (83.21–161.63)	111.07 (83.41–152.35)	0.257
< 130	106 (70.76)	85 (56.67)	191 (63.67)	0.012*
≥ 130	44 (29.33)	65 (43.33)	109 (36.33)	
HbA1c (%)	6 (5.7–6.4)	6.9 (6.2–8.5)	6.35 (5.9–7.9)	< 0.001*
Fasting blood sugar (mg/dL)	100 (94–128)	139 (112–194)	119.5 (97.5–161.5)	< 0.001*
Triglyceride (mg/dL)	113 (75–151)	112 (82–158)	113 (81–151)	0.696
Total Cholesterol (mg/dL)	144.5 (124.5–168)	144 (116–173)	144 (122–172)	0.623
LDL‐C (mg/dL)	81 (65–106)	93 (66.5–120)	87 (65–115)	0.183
HDL‐C (mg/dL)	37 (32–43)	34 (31–43)	35 (31–43)	0.267

Data presented as median and interquartile range (Q1–Q3) or frequency and percentage

* statistically significant, *P*‐value < 0.05

Regarding PLR, the median value for all participants was 111.07, with an IQR of 83.41–152.35. After grouping the PLR values based on Youden's index, 43.33% of patients with 3VD and 29.33% of individuals with normal coronary arteries were in the elevated PLR group (PLR ≥ 130). This difference in distribution was also statistically significant (*p* = 0.012). Further analysis and comparison of other clinical parameters are reported in Table 2.

### 3.3 Correlation of NLR and PLR with Cardiac Function

**Figure 2:**
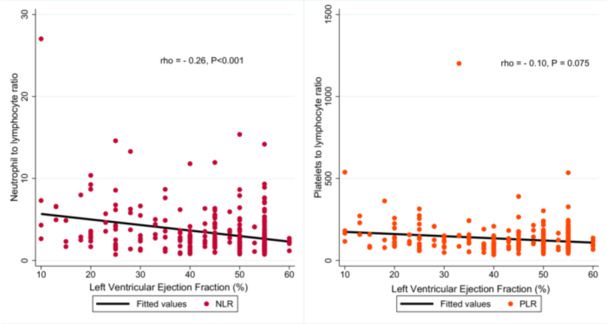
Correlation of NLR and PLR with left ventricular ejection fraction.

Further analysis showed an inverse correlation between NLR and left ventricular ejection fractions (LVEF) (rho = −0.26, *p* < 0.001), indicating that higher NLR values are related with decreased cardiac function. While PLR showed a similar result, this correlation with LVEF was weaker and did not reach statistical significance (rho = −0.10, *p* = 0.075) (Figure 2).

### 3.4 NLR and PLR and Type of Involved Vessels

According to the results in Table 3, the type of vessel involved was examined based on NLR and PLR values in individuals with severe coronary artery disease (*N* = 150). The most common vessels involved in these patients were the RCA (91.33%), LAD (89.33%), and LCX (71.33%), respectively. After comparing the type of vessel involved based on NLR and PLR, it was observed that 16.67% of individuals with elevated NLR (compared to 8.33% in the group with NLR < 2.5) and 15.38% of individuals with elevated PLR (compared to 11.76% in the group with PLR < 130) had severe involvement in the left main coronary artery, which is one of the most important coronary vessels. However, this difference in distribution was not statistically significant (*p* > 0.05). Details of the related information are reported in Table 3.

**Table 3:** Comparing the type of vessel involved in three vessel coronary artery disease (3VD) patients between NLR and PLR categories.

Type of vessel involved	NLR < 2.5 (*n* = 60, 40%)	NLR ≥ 2.5 (*n* = 90, 60%)	*P*‐value	PLR < 130 (*n* = 85, 56.67%)	PLR ≥ 130 (*n* = 65, 43.33%)	*P*‐value	Total (*n* = 150)
Left main	5 (8.33)	15 (16.67)	0.141	10 (11.76)	10 (15.38)	0.518	20 (13.33)
Proximal LAD	6 (10.0)	11 (12.22)	0.674	10 (11.76)	7 (10.77)	0.849	17 (11.33)
LAD	54 (90.0)	80 (88.89)	0.829	77 (90.59)	57 (87.69)	0.569	134 (89.33)
Diagonal	35 (58.33)	47 (52.22)	0.461	49 (57.65)	33 (50.77)	0.402	82 (54.67)
LCX	41 (68.33)	66 (73.33)	0.507	61 (71.76)	46 (70.77)	0.894	107 (71.33)
OM	41 (68.33)	65 (72.22)	0.608	58 (68.24)	48 (73.85)	0.455	106 (70.67)
Ramus	3 (5.0)	9 (10.0)	0.363	6 (7.06)	6 (9.23)	0.627	12 (8.0)
RCA	55 (91.67)	82 (91.11)	0.906	78 (91.76)	59 (90.77)	0.830	137 (91.33)
PDA	8 (13.33)	20 (22.22)	0.171	12 (14.12)	16 (24.62)	0.102	28 (18.67)
PLV	2 (3.33)	9 (10.0)	0.201	3 (3.53)	8 (12.31)	0.057	11 (7.33)
SVG	3 (5.0)	6 (6.67)	0.742	7 (8.24)	2 (3.08)	0.300	9 (6.0)
LIMA	1 (1.67)	3 (3.33)	0.650	1 (1.18)	3 (4.62)	0.317	4 (2.67)

Data presented as frequency and percentage (%)

### 3.5 Association of NLR and PLR With Three‐Vessel Disease

The results of univariate and multivariable models predicting 3VD, stratified by NLR and PLR factors, are shown in Table 4. After independently examining both NLR and PLR in relation to the 3VD, both factors were found to have a significant association with the 3VD at the univariate level (*p* < 0.05).

**Table 4:** Results of univariate and multivariable logistic regression about association of NLR and PLR with three vessel coronary artery disease (3VD).

Factor		Model 1 Crude OR^1^, 95% CI	*P*‐value	Model 2 Adjusted OR, 95% CI	*P*‐value	Model 3 Adjusted OR, 95% CI	*P*‐value
**NLR**	< 2.5	Reference	0.001*	Reference	0.018*	Reference	0.037*
	≥ 2.5	2.25 (1.41–3.57)		1.88 (1.11–3.18)		1.91 (1.04–3.51)	
**PLR**	< 130	Reference	0.012*	Reference	0.102	Reference	0.077
	≥ 130	1.84 (1.14–2.96)		1.58 (0.91–2.74)		1.79 (0.93–3.41)	

Model 1: **NLR or PLR**

Model 2: gender, age, **NLR or PLR**

Model3: gender, age, **NLR or PLR**, Waist‐to‐hip ratio, history of diabetes, dyslipidemia, hypertension, cancer, chronic kidney diseases, smoking, drinking alcohol, family history of IHD, history of anti‐diabetics agents/insulin, lipid‐lowering agents, and anti‐hypertensive agents.

* statistically significant, *P*‐value < 0.05.

^1^ Odds ratio, 95% Confidence Interval.

After considering the effect of other probable confounding variables on the 3VD, it was observed that individuals with NLR ≥ 2.5 had a significantly increased odds of 3VD by 91% compared to individuals with NLR < 2.5 (OR = 1.91, 95% CI = 1.04–3.51, *p* = 0.037).

Furthermore, after separately examining the predictive effect of PLR on the 3VD, it was also observed that, after considering confounding variables, individuals with PLR ≥ 130 had 79% increased odds of 3VD compared to individuals with PLR < 130, although these results were not statistically significant (OR = 1.79, 95% CI = 0.93–3.41, *p* = 0.077) (Table 4).

### 3.6 Model Performance and Validity Indices

Table 5 presents the validation indices of statistical models. The validation indices indicate that the models have good validity and diagnostic ability based on the values of sensitivity, specificity, positive and negative predictive values, and accuracy, with a 50% prevalence of 3VD in this study. Ultimately, the accuracy of correctly predicting the outcome in the NLR statistical model was estimated to be 78.38%, and in the PLR model, 79.39%.

**Table 5:** Predictive value of the adjusted NLR and PLR to predict three vessel coronary artery disease (3VD) based on multivariable Logistic regression results.

Indices	NLR	PLR
	Values^1^	Values^1^
Validity indices		
True positive (*n*)	122	122
True negative (*n*)	110	113
False positive (*n*)	36	33
False negative (*n*)	28	28
Sensitivity (%)	81.33	81.33
Specificity (%)	75.34	77.40
Positive Predictive Value (PPV) (%)	77.22	78.71
Negative Predictive Value (NPV) (%)	79.71	80.14
Accuracy (%)	78.38	79.39
Discrimination index		
AUC (95% confidence interval)	0.85 (0.81–0.89)	0.85 (0.81–0.89)
Calibration index		
2H–L tests, X^2^(P)	5.11 (0.745)	5.29 (0.725)

^1^ Validity indices estimated according to the prevalence of 3VD in our study (Prevalence = 50%).

^2^ Hosmer–Lemeshow's test.

In addition, the area under the curve (AUC) as a discrimination index of the model, for the predictive effect of NLR and PLR, adjusted for the effect of confounding variables on the occurrence of 3VD, was 85% (AUC = 0.85, 95% CI = 0.81–0.89). The obtained values indicate that the statistical model has good discrimination ability.

Furthermore, after examining the calibration of the statistical models using the Hosmer‐Lemeshow test (in terms of agreement between the observed outcome and the outcome predicted by the models), it was observed that both models were favorable with respect to the indices considered (*p* > 0.05) (Table 5).

## 4 Discussion

This study evaluated the role of NLR and PLR as predictive biomarkers for three‐vessel coronary artery disease (3VD) in patients undergoing elective coronary angiography (CAG). Our findings demonstrated that elevated NLR values (≥ 2.5) were significantly associated with 3VD. Additionally, PLR was identified as a potential risk factor for 3VD, but this association was not statistically significant after adjustment for confounders.

The association between NLR and CAD severity (3VD) aligns with previous studies. Sari et al. (2015) reported that higher NLR and PLR values were significantly associated with CAD severity, though our study had a larger sample size (300 vs. 180) and demonstrated higher predictive accuracy for NLR (AUC = 85% vs. 72.6%) [1]. Additionally, our study established that NLR ≥ 2.5 increased the odds of 3VD by 91% (*p* = 0.037), reinforcing its clinical significance.

Our results are also in concordance with the findings of Gordhanbhaie et al. [12] (2023), who similarly identified NLR as an independent predictor of CAD severity. However, key differences exist: Our study included a control group, whereas Gordhanbhaie et al. did not. The NLR threshold differed, with our study establishing NLR ≥ 2.5, while their study suggested NLR ≥ 2.15.

In general, NLR and PLR can be effective markers of systemic inflammation and the immune‐inflammatory response in atherosclerosis [23]. The plausible biological basis for this association lies in the dual components of the NLR. Neutrophils, as first responders of innate immunity, are major contributors to vascular inflammation [24]. They secrete proteolytic enzymes, reactive oxygen species (ROS), and pro‐inflammatory cytokines that promote endothelial dysfunction, plaque destabilization, and thrombosis. Elevated neutrophil counts have been associated with greater plaque burden and vulnerability in both experimental and clinical studies [24, 25]. Conversely, lymphocytes, especially regulatory T cells, play a modulatory and protective role by suppressing excessive immune activation and maintaining vascular homeostasis [26]. A reduced lymphocyte count may reflect impaired adaptive immune regulation or heightened physiological stress, both of which are associated with adverse cardiovascular outcomes [27]. Thus, an elevated NLR represents a state of heightened innate immunity and suppressed adaptive response, which synergistically contribute to the progression and severity of atherosclerosis [28].

Several previous studies have supported the prognostic significance of NLR in CAD. For instance, available studies have demonstrated a strong association between high NLR and cardiovascular mortality [29, 30], while a recent meta‐analysis has confirmed its predictive value for both disease severity and long‐term outcomes [31]. Our findings add to this growing body of evidence, specifically emphasizing NLR's relevance in identifying patients with advanced, multi‐vessel coronary involvement.

Although PLR was also elevated in patients with 3VD, it did not maintain statistical significance after multivariable adjustment. Similarly, Akboga et al. (2016) demonstrated that PLR was correlated with CAD severity, using the Gensini score as a classification method [17]. This discrepancy may be attributed to differences in population characteristics, sample sizes, or additional confounding factors.

While PLR reflects platelet activation and systemic inflammation, it may be less specific or sensitive than NLR in the context of coronary disease severity. Heterogeneity in the inflammatory response, platelet turnover, and study methodologies may account for the inconsistent findings related to PLR in the literature [17, 32].

This study identifies as the current evidence, that NLR as a simple, cost‐effective, and non‐invasive biomarker [33] for predicting 3VD, underscoring its practical utility for early risk stratification, especially in resource‐limited settings. While PLR did not show a statistically significant independent association, its recognized role in systemic inflammation suggests potential value as part of a multi‐biomarker panel in future research.

### 4.1 Limitations and Future Directions

Despite the strengths of this study, several limitations must be acknowledged:

First, its case‐control design may introduce selection bias. Second, the limited sample size precluded internal validation via a train‐test split. Third, we did not incorporate established angiographic risk scores (e.g., Syntax or Gensini scores) for comparative analysis. Fourth, as a single‐center study in Iran, the findings may not be generalizable to other populations. Finally, despite adjusting for major cardiovascular risk factors, residual confounding from unmeasured variables (e.g., genetic factors, other comorbidities and acute or chronic infectious or inflammatory diseases) is possible.

Future prospective, multi‐center studies with larger cohorts are needed to validate our findings, assess the prognostic value of NLR and PLR, and evaluate their applicability across diverse ethnic and clinical settings.

## 5 Conclusion

This study provides strong evidence supporting NLR as an independent predictor of severe CAD, with an NLR threshold of ≥ 2.5 demonstrating high accuracy for three‐vessel disease detection. PLR, while associated with CAD severity, did not retain statistical significance after adjusting for confounders. Given the low cost and accessibility of NLR, its integration into routine cardiovascular assessments may enhance early detection and risk stratification of CAD.

Future studies should explore additional inflammatory markers and risk prediction models to optimize CAD screening and management.

## Author Contributions

**Niloufar Taherpour:** methodology; formal analysis, software, data curation, writing – review and editing.

## Funding

The authors received no specific funding for this work.

## Disclosure

The authors have nothing to report.

## Ethics Statement

Ethical approval has been granted by the Deputy for Research Affairs, Shahid‐Beheshti University of Medical Sciences (IR. SBMU. RETECH. REC.1401.874). In addition, ChatGPT (GPT‐5.2) was used solely for language editing and did not contribute to the scientific content of the manuscript.

## Consent

The authors have nothing to report.

## Conflicts of Interest

The authors declare that they have no conflicts of interest to report.

## Transparency Statement

The lead author, Niloufar Taherpour, affirms that this manuscript is an honest, accurate, and transparent account of the study being reported; that no important aspects of the study have been omitted; and that any discrepancies from the study as planned (and, if relevant, registered) have been explained.

## Data Availability

The data that support the findings of this study are not publicly available. However, data are available from the corresponding author upon reasonable request and with permission of the ethics committee of the deputy of research and technology in Shahid Beheshti University of Medical Sciences.
